# The intersection of relational autonomy and narrative ethics for the patient unwilling to disclose genetic diagnosis information

**DOI:** 10.1186/s40504-014-0007-6

**Published:** 2014-03-18

**Authors:** Michael Gallagher

**Affiliations:** Department of Health Care Ethics, St. Joseph’s University, 5600 City Avenue, Philadelphia, PA 19131 USA

## Abstract

The rare case of the patient unwilling to disclose genetic data to his or her family provides an opportunity to expand the atomistic conception of the autonomous individual in medical decision-making. Medical practitioners naturally avoid violating patient autonomy and privacy. However, unwilling disclosure can damage the health of people other than the patient. In this situation, professionals must weigh the principle of autonomy against the nature of relationships, duties, and confidentialities between patient, professional, and family. The paradigm case studied is that of a patient with a potentially dangerous heart condition, Long QT Syndrome 3. Patients with Long QT 3 are at high risk for dying of ventricular tachycardia during rest, especially from ages 40–60. Once familial genetic testing was completed, the proband's mother, who was positive for the mutation, chose not to inform her estranged sister of the diagnosis.

This paper examines the ethical duties of the physician to inform a patient's extended family of a serious genetic diagnosis, with a focus on the emotional and psychological effects of genetic testing. The need to adapt the process of violating confidentiality around considerations for the patient's emotional state and narrative will be addressed. This approach considers the patient’s narrative, standpoint, and relationships as a way to develop a support plan and will present a guideline for cases where the probability of significant harm to others supersedes the patient’s preference of non-disclosure as well as the physician’s respect of confidentiality. The paper seeks to expand the conversation on genetic testing and autonomy beyond principles by considering all parties involved and emphasizes the use of the varied resources available to medical practitioners, especially to provide the best help possible without overburdening physicians with duties.

## Case study

The proband, a 20-year-old male soccer player asymptomatic for cardiac illness, presents with an abnormal EKG at a routine physical. A second EKG confirms a prolonged QT interval, which is associated with an increased risk of sudden cardiac death. However, given that the proband played soccer for many years with no symptoms, his doctor reassures him that a positive diagnosis is unlikely. Genetic testing results in a positive diagnosis of inherited long QT syndrome type III (LQT3). An electrical disorder of the heart caused by a mutation of cardiac ion channels, LQT3 can cause a ventricular arrhythmia called torsades de pointes (TdP). TdP produces palpitations, fainting, and can potentially cause sudden cardiac death due to ventricular fibrillation. Different types of Long QT syndrome (LQTS) produce TdP at different times. For example, patients with LQT3 are most at risk during sleep, especially between ages 40–60. LQT3 is among the most lethal forms of the disorder, but it is not particularly common, striking around 10% of sufferers (NIH 2011). Other forms of LQTS can cause arrhythmia during exercise or during high stress moments.

The proband’s family was referred for genetic counseling. Familial testing determined that the proband’s mother was the carrier. The proband’s sister and father were negative for the mutation. The mother was then called in by her physician to determine how to best inform family members. The mother told the physician that she had only a sister with two children living in Florida. Both of the mother’s parents had passed away, one due to stroke at age 64 and the other due to pneumonia at age 81.

When the doctor urged the mother to inform her sister, she replied, “Absolutely not. I’m not calling her.” The physician offered a number of other modes of communication, including mailing forms himself, but the mother stated that she did not want to open any line of dialogue with the estranged sister. She said, “She would try to talk to me. I don’t want to talk to her.” When the physician asked what might be causing these negative feelings, the patient was unreceptive.

Given the risk of death for the estranged sister, the physician felt his hands were tied. On one hand, the patient did not have a definitive, life-threatening illness. Rather, the odds of dying from undiagnosed LQT3 are roughly 50% (NIH 2011). The physician did not feel the duty to warn would apply from a legal or an ethical standpoint, but also felt a strong desire to make sure this unknown woman knew the potential life changes necessary to make sure her risk of death was minimized. However, due to the legal concerns surrounding the case, the physician erred on the side of safety and followed the wishes of the proband’s mother. He turned to the American Medical Association (AMA 2004) guidelines and found he had a minimal duty to inform the patient of her risk. He left her with the advice, “You should inform your entire family.”

## Background

The risk for death for some undetected forms of LQTS can be serious. All forms of LQTS have a death rate of approximately 9%, and LQT3 itself is the most lethal type of the disease (NIH National Institutes of Health 2011). For LQT3, the largest risk factors outside of general periods of rest are being an endurance athlete (resting heart rate under 60 bpm) and taking drugs which induce QT prolongation (*ibid*). The list of QT-prolonging drugs is vast, including most antihistamines and decongestants, diuretics, statins, antidepressants, and many common antibiotics (NIH).

Generally, LQTS is treated through the use of beta blockers. However, where other forms of LQTS worsen when the heart rate increases, LQT3 becomes worse when the heartbeat slows. Therefore, LQT3 does not respond well to beta blockers. Patients with LQT3 can regulate cardiac ion concentrations by supplementing their diet with sodium and potassium, but the only medical intervention available is the use of an implantable cardiac defibrillator (ICD) at the onset of symptoms.

Case law sets precedent to allow clinicians to warn parties outside the physician-patient relationship if a patient intends harm to himself or others. The duty to warn was established by the California Supreme Court's ruling in *Tarasoff v. University of California.* The case was brought to the court by the Tarasoff family, whose daughter Tatiana had been stalked and murdered by a psychiatric patient at the University of California Berkeley, Prosenjit Poddar. The court ruled that a clinician has a duty to not only her patients, but society at large. If the patient intends to harm himself or others, confidentiality should be breached in order to protect that patient and anyone she might harm (17 Cal. 3d 425). That is, the harm incurred by breaching physician-patient confidentiality is less than the harm potentially incurred by the patient to himself or to society.

Later case law is divided on other cases of duty to warn. Some states have statutes that require a physician to warn her patients’ next of kin if the patient has HIV and intends to engage in unprotected sex or share needles (Worth et al. 2008). In *Pate v. Threlkel,* Heidi Pate sued her mother’s physician because he did not warn Ms. Pate of her mother’s hereditary medullary thyroid cancer (MEN). Like LQTS, MEN is inherited in an autosomal dominant pattern. Early diagnosis of MEN can allow for life-saving intervention, but in Ms. Pate’s case, she was found to have advanced thyroid cancer three years after her mother’s diagnosis. She filed suit, alleging that if the physician had warned her of her mother’s genetic diagnosis early enough, Ms. Pate's disease progress could have been halted (Offit et al. 2004). Essentially, the court decided that the standard of care sometimes is written to the benefit of third parties (662 Fla.). However, the court also declared the duty of the physician fulfilled by warning the patient, not the family. While the duty to warn extends to third parties, it is not required to inform them. Two cases in New Jersey and New York, *Safer v. Peck* and *Tenuto v. Lederle Laboratories,* extend the duty to warn immediate family members of risky hereditary conditions and services like vaccinations which may incur harm to unimmunized family members (90 NY2d 606).

The duty to rescue is a concept developed in tort law. Duty to rescue exists in two situations: first, when one party creates a situation that is dangerous for another party; second, when a party has a “special relationship” to another, such as a parent to a child or spouses to each other (545 US 748). While case law does not extend the duty to rescue to siblings, it provides an interesting legal concept for the case. First, the mother may have developed a situation that is dangerous for her sister – not informing the sister of a diagnosis that kills around one in ten of its sufferers. Second, her blood-relatedness seems to imply a special relationship at least similar to that of the fiduciary relationship owed by a physician to her patient. As a legal concept, duty to rescue has a limited application – but when considered in the sense that one can owe a duty of rescue to a person of close blood-relatedness, may have value in an ethical analysis.

While some state courts extend a vague duty to warn immediate family members with potential risk, physicians and professional societies remain more cautious. The AMA (2007), American Society of Human Genetics, and American Society of Clinical Oncology agree on some variation of the AMA policy: physicians should inform patients of circumstances under which confidentiality would be breached and to “make themselves available to assist patients in communicating with relatives to discuss opportunities for counseling and testing, as appropriate,” but that duties are fulfilled by disclosing genetic results to the patient alone. Physicians tend to follow this rule - a survey of 800 practicing or formerly practicing geneticists showed that only 23% of the sample would be willing to disclose information to a patient’s family even if the risk to the family member was high (Falk et al. 2003).

LQTS inheritance probability is generally 50% (NIH 2011). In the case of violent psychiatric patients, terminal genetic conditions like Tay-Sachs or Huntington’s, or even some HIV disclosure cases, doctors are often more aware of impending and largely definite harm than in genetic predispositions like LQTS. In a purely practical sense, a physician would need to parse out a number of different rules in order to determine whether a disclosure is necessary. The duty of care in this case would depend on estimated risk to a given family member. Physicians naturally exercise caution when the patient is unwilling to disclose to at-risk relatives. The dichotomy between the law and actual practice indicates the difficulties inherent to decision-making about the disclosure of genetic dispositions. While physicians may not be required to actively lie or withhold information, as might be the case with exercising therapeutic privilege for a cancer diagnosis to a very optimistic but fragile patient, they can be inclined by the standard of care not to inform at-risk people. The physician can violate the basic respect for the autonomy of his patient to potentially save another life, or fulfill the minimal responsibility to his patient but allow the other life to hang in the balance.

While the traditional normative ethical theories, such as principlism or utilitarianism, might be useful in less complex situations, there are clearly multiple conflicts of basic principles and parties here. Beauchamp and Childress (2001) write that autonomy serves as a "right, not a duty of patients.” That is, the patient's right to make decisions for a treatment course must be protected by all parties but that autonomous decision-making is not demanded in all cases. Moreover, the two authors argue the professional’s obligation to the patient is "respectful treatment in disclosing information and fostering autonomous decision-making" (*ibid).* This conception of autonomous action empowers the individual to make decisions and avoids medical parentalism. Balancing principles can be difficult without a view into the reasoning of both parties in a conflict. The mother's autonomy and trust in the physician-patient relationship is significantly diminished by disclosure, but a life is potentially saved. Physicians also possess a duty to warn in order to avoid deliberately allowing a harm otherwise preventable with genetic testing information. Yet probability remains a factor that renders principles difficult to use - the sister not have the disease, may never present symptoms, or may die.

Feminist and narrative methods remind the ethicist to find a degree of understanding with the perspective of the patient. Both theories tend to focus on humanizing the parties involved in ethical dilemmas, rather than applying rigid rules or principles to a situation. On the other hand, narrative and feminist ethics tend to provide very vague or even no solutions to ethical dilemmas. Questions of a person's life story, standpoint, or autonomy in relation to others are still arguably best served by these methods. By obtaining a picture of the whole person, narrative ethics can shift the focus of disclosure dilemmas from violating rules or duties to changing perceptions. Alternative methods to traditional normative means supplement the principlist conception of autonomy with relational aspects, balancing interconnectedness rather than simple individual action.

### Emotional and relational issues

The nature of the patient's emotions and relationships can determine the extent to which the patient is willing to disclose. At the outset of a genetic diagnosis, emotions tend to run high but wane with time. Aatre and Day (2011) document a number of emotional issues arising from inherited cardiovascular diseases, ranging from reassurance to outright fear. Interview studies have shown that genetic testing can leave a patient feeling devastated. One patient said, “I was thinking, what other genes are also defective? [. . .] I also wanted to take on a new identity” (Porz 2009). Genetic testing results produce issues of identity crises because they make people feel as if they are no longer self-governing. Patients report feeling “powerless, disoriented, confused” (Porz 2009). The body can now be seen to harbor dangerous genetic flaws or defects and the patient is reminded strongly of their own mortality. The mother in the case study may have felt similarly adrift, and also may have dealt with guilt over passing the disease on to her son. Quality of life may be adversely affected, further adding to questions of her future.

Looking at the standpoint of the mother in the case study, she likely feels some degree of guilt over passing this disease on to her child. As a 20-year-old soccer player, her son might be denied the chance to continue playing his sport, or any contraindicated competitive sport, should he inform anyone of his diagnosis. Even without symptoms, a positive LQTS diagnosis is typically enough for a physician to recommend against competitive sports Pelliccia et al. (2005). The mother may be transferring the role of the denier to herself, allowing herself to feel as if "her" disease has guaranteed the son's loss of autonomy. Since the proband was asymptomatic, the mother might feel as if there is no risk to her sister, that nothing truly wrong with her son, and may even mistrust the diagnosis. She could therefore justify nondisclosure by the increasingly present reality of probabilities in her life, thinking, "My son had only a 50% chance of getting this from me, my daughter did not get it, and I only had a 50% chance of getting it from my mother." Taken together, the mother may feel that disclosure is unnecessary because her sister simply does not seem to be under any realistic risk.

Another valuable consideration in the case of such significantly diminished autonomy is the idea of control (Aatre and Day 2011). Inherited diseases wrest the power over one's body from the individual and place it in the hands of chance. The establishment of control could be expressed through a number of outlets, including "self-education, maintaining privacy, and active participation in treatment decisions" (*ibid*). Maintenance of privacy speaks to the case study, where the mother may be seeking to keep secrecy surrounding her condition. Secrecy can be a method of control, as it allows the patient to determine with whom she discusses the diagnosis. The emotional nature of disease can be a difficult subject to broach for people with whom one is uncomfortable, especially when the diagnosis is potentially life-threatening. While legal precedent argues for a duty to warn and the right-to-know, patients have their own perceptions of that right to know. A patient may feel that a genetic diagnosis is his or hers alone, not focusing on the importance of extending that diagnosis to his immediate family.

The desire to obtain control in a situation of diminished autonomy can also be tied to the establishment of relational dynamics. Feminist ethics can be useful in examining how relational dynamics affect the situation. Nodding's care ethics provides a relatively simple definition of what variations on relationships exist in the case. For example, the physician-patient relationship would be described as a "caring-for" relationship where the face-to-face encounters between the one-caring (physician) and cared-for (patient) create a direct relationship (2001). The indirect relationship between the physician and the patient's sister seems closer to "caring-about," which Noddings identifies as having a "benign neglect" (2002). However, caring-about is somewhat foundational, establishing the basics for caring-for and a general sense of social justice.

The justice-as-fairness derived from caring-about can help explain why the physician feels conflicted over the disclosure case. His sense of justice tells him it is fair for the sister to know information that can affect her future, but directly violating the patient's autonomy seems a greater offense than indirectly and only potentially harming an unfamiliar outsider. With the knowledge granted by the mother's genetic test, the physician can prevent a potential harm. It is this sort of more egalitarian sense of weighed duties that causes problems for a physician. Either perspective, respect for the mother’s autonomy or nonmaleficence towards her sister, places the burden on deciding in favor of a single party. The desire to create a fair, just reality for both people establishes the conflict and is arguably impossible to satisfy with an ethic that focuses purely on individuals and not on relational communities.

Arguably, the mother is marginalizing her sister by denying her sister access to the reality of her genetic illness. This ties into the concept of causal relational autonomy, where an outside factor (the mother) reduces the autonomy of a moral agent (the sister). One formulation of this version of autonomy involves a theory of "significant options" available to an autonomous agent at the time of a decision (Brison 2000). That is, an agent must have the proper grasp of all external factors in order to have the options necessary for a decision. The sister may be acting in an entirely autonomous manner, but her decisions could be altered by the knowledge that she has a genetic illness. On one hand, an LQTS diagnosis might constrain her actions in a more significant way than not having a diagnosis would. However, the proband's mother has knowledge that prevents her sister from making a fully *informed* decision. The sister lacks all available options - for example, she could choose not to run a marathon because doing so might put her at risk of arrhythmia. By knowingly withholding key information, the mother reduces her sister's ability to make choices about her lifestyle and the lifestyles of her children. Feminist theorist Annette Baier argues, "persons are essentially successors, heirs to other persons who formed and cared for them" (Baier 1985). That is, the patient's caring-for her sister influences how her sister can exercise autonomy. The sister may be an autonomous agent without knowledge of her genetic illness, but the mother has tools to allow her sister a deeper knowledge of the risks involved in her day-to-day activities. In essence, the broken social relationship between the proband's mother and her sister has reduced the sister's ability to make informed choices.

Obviously, these considerations place strain on the principle of individual autonomy. However, it is arguably the focus on autonomy in modern medical ethics which creates the conflict in this case. Feminist theorists recognize that autonomy develops from a conflation of external influences - from personal relationships to the social framework a person inhabits. This context for autonomy reminds the ethicist that free decisions come from a personal narrative, influenced by the encounters and experiences of life. In essence, the autonomous individual cannot separate himself from the outside community in any way. While the case study may not merit a violation of autonomy through disclosure, it does remind the ethicist that medicine cannot always concern itself solely with the individual patient and professional. Other parties are almost invariably involved, be it the impoverished person who might be harmed by improper resource allocation or the sibling whose well-being is threatened by a nondisclosure. Moreover, human reason can be fallible, especially with regard to the future (Levy, 2011). Perhaps a trajectory towards a more communitarian ethic, based in the relatedness of people through their interactions and social development, is needed.

Medical ethics adapted libertarian concepts of individuality to protect patients from professionals who held complete power. In doing so, however, medicine tipped the balance too far by ignoring the possibility of constraining patient autonomy. The dynamic fostered by a communitarian ethic would ideally be one of support and understanding, where the medical team is responsible for providing care to a patient in interaction with that team. The current dynamic fosters much advice-giving on the part of the medical team and much decision-making for the patient, but rarely do the two meet as equals. Suggestions for care could be made by both parties and considered with medical expertise and patient values in mind, but a balance could be struck between the patient and physician’s empowerment.

Rouven Porz attempts to adapt Monica Konrad’s “kinship ethics” to situations similar to this one, arguing that the principles valued by medical ethics are insufficient for family members struggling with genetic data. One important principle Porz emphasizes is the idea of loyalty to family members and the relatedness of the human species. Genetic testing unites a person with a larger web of the “new genetic family,” the sort of extended family network developed through awareness of a genetic disease (Porz 2009). “Genetic constitution,” not blood relatedness, determines the interrelatedness of the genetic family (*ibid*). Therefore, genetic disclosures can become an issue of loyalty between members of a community composed of more than private individuals or separate unit of blood relatives. Altruism can be one way a family member fulfills this loyalty - the outright giving-away of genetic information. However, for more distant genetic relatives, that sense of altruism may not be present. Kinship offers a second alternative: reciprocity. The reciprocal sharing relationship between two people provides a secondary outlet for genetic information. The narrative of genetic interconnection expands responsibilities of sharing to a larger community through a transformation of the personal narrative.

Kinship theory removes the feeling of a patient’s ownership of their genetic information by establishing the idea that a given mutation is not unique. Rather, the patient is part of a continuing family narrative, reaching into the past and potentially extending into the future. The owed debt to this family means that while the mother in the case study can withhold information about her treatment for Long QT, she cannot withhold information about the family having a genetic history which predisposes its members to LQTS. This conception of the narrative can provide the physician with a way to frame the situation for the mother. Consider, for example, the idea of duty to rescue for someone with an understanding of kinship ethics. As one’s genetic network expands, the duty to rescue can be owed to a number of genetic relations – family members at risk for inheriting a disease. Combined with the obligation of rescue or the strength of altruistic intentions, kinship ethics can become a valuable determining factor for disclosure practices.

### Suggestions for practice

Knowledge of the different ways in which one's life story can be interpreted is useless without a way to inform the patient of these new concepts. Patient education is one of the most commonly used parts of dealing with difficult issues in the doctor's office. Yet a study of patients with hereditary colorectal cancer, who used educational materials like letters and booklets to understand their disease, did not show significant differences in knowledge compared to control group without the additional material (Gaff et al. 2005). Education after the fact may not be useful. Additionally, legal precedent and professional attitudes conflict. The principle of doctor-patient confidentiality presents an ethical reason not to disclose, and geneticists often feel bound by the limited code of the American Society of Human Genetics, but many are unaware of the professional code (Falk et al. 2003). Even those physicians with knowledge of professional codes report their duty is typically to inform the patient, and no other duty is required to next-of-kin (*ibid*).

The general tendency to avoid disclosure points to a significant valuation of privacy and consent. Therefore, a novel approach for reopening communication might be the establishment of an alternative narrative. The concept of the kinship narrative has already shown such an alternative conception is possible. However, the new narrative is only valuable if it can be used to facilitate communication between at-risk relatives. If providing educational material fails the patient, then perhaps perceptual or behavioral changes succeed.

Providing this sort of perceptual change can seem difficult for one physician to accomplish. The increasing awareness of fragmented care in the medical field has led to much pressure on the physician to improve her practice by becoming a sort of scion of morality, competence, and altruism, a Renaissance man operating with ever-limited time for each patient. The duty to contact and warn an estranged relative, as in the case study, might be seen as an example of this. Steel (2009) presents a similar example - the patient does not want to disclose a life-threatening diagnosis to his estranged cousin in Australia. The physician obviously cannot spend the time finding contact information for this remote cousin without cooperation from the patient. Even in less drastic situations, the physician needs another party to carry some of the burden.

A practical suggestion would be to place responsibility in the hands of other health care services. A patient struggling with the emotions of a genetic diagnosis or disclosure can be referred to therapeutic counseling. There, a professional can provide the necessary tools to explore the issues the patient may have with both the disease and the relationship with her sister. Obviously, therapy sessions require that a patient accept the idea of going to therapy as a net good. However, counseling has been offered for an increasing number of conditions which might require significant lifestyle adaptation for a patient. There is no reason to avoid working through the emotions of someone with a difficult diagnosis, no matter what form that diagnosis takes. If counseling should succeed in making the patient more comfortable with disclosure, then the geneticist can go forward with referring at-risk family members.

A second route for a physician might be an outright breach of confidentiality. Concerns for autonomy and the physician-patient relationship mean this should be a last resort reserved for extreme situations, but a threshold of likelihoods across which disclosure would be permissible can be useful. In any case, respect for autonomy means that a significant burden of proof is placed upon a geneticist breaching confidentiality and that the patient must be informed of a last-resort policy before any testing occurs, in accordance with the AMA’s policy recommendations.

This decision-making process does not come without its risks. Since genes only convey probabilities in many situations, decision-making cannot be reduced to seeing if a gene is present or not. For LQT3 patients, the presence of the gene only conveys a probability of passing it along to descendants and an additional probability of cardiac issues. The developing theory of epigenetics offers hope that the field will steer away from reductionism by emphasizing the effects of the environment and other non-gene directors of genetic expression. However, in making decisions to weigh *potential* inheritance and *potential* harm, physicians need to be careful that they rely on realistic risk and not simply knowledge that the gene is present in a patient. Not all genes in a given patient will penetrate to all family members, and even penetrant genes may not be expressed or produce symptoms. To force the mother to disclose simply because the LQT3 gene was present in her body would border on discrimination. Adequate weighing of risk and benefit must come before a disclosure decision.

Disclosures might be best when the following criteria are met:

1. the at-risk family member can be contacted,
2. the illness has a high chance of inheritance (greater or equal to 50%),
3. the illness will eventually be serious and life-threatening,
4. interventions (treatment or lifestyle changes) can cure or significantly reduce the effects of the disease,
5. the patient is adamant with regard to non-disclosure

These considerations outweigh the potential harm to the proband's autonomy because a significant preventable harm can be overcome by the violation of confidentiality, similar to the legal right to the duty to warn enshrined after *Tarasoff*. On one hand, the proband might be denied the sense of control he desires. However, the uninformed at-risk party would be denied knowledge which could prevent greater losses, such as preventable debilitation or death.

If applied to the case under analysis, such criteria would likely not be enough to justify a disclosure because the likelihood of death is relatively low. Moreover, no intervention short of an ICD could affect the disease course. Given that ICDs are generally only implanted at the onset of symptoms, the most that could be done for the sister would be reducing participation in strenuous competitive sports and perhaps changing the diet. There exists little reason to breach confidentiality here, but other cases would certainly merit this saving grace. What, then, can be done for the mother? Certainly counseling sessions can be offered, as her life has changed drastically and she seems to be struggling with family connectedness. Time may be the only way to reach a reasonable disclosure – however, unilateral decision-making on the part of the physician would represent both a gross violation of patient rights and a discriminatory act.

The sister in the case might act differently if she knows she suffers from LQTS, and the focus on autonomy in medical practice neglects that potentiality by valuing only the decision-making of the person in front of the doctor. Yet narrative and relational methods rely on just these sort of connections by recognizing the value of interpersonal effects on decision-making. While a libertarian ethic of medicine protects the individual patient from the individual practitioner, it fails to reflect on the full scope of decisions and harms caused to outside parties by overvaluing singular moral agents.

Principlism has succeeded in providing medical ethics with a basis around which to develop patient rights but has arguably failed in adequately ensuring a humanistic dialogue between physician and patient by overemphasizing autonomy. However, principlism need not be discarded, as it offers helpful points on which case decisions can be made. Rather, it needs to be supplemented with a sense of humanity and a respect for the life narrative of the patient and physician, reminding both of their indebtedness to society and those who helped them weave those narratives. Autonomy should not be considered "first" among equals, but rather one of many goals towards which good medical practice strives. People are not merely gaseous molecules, sometimes brushing past each other but otherwise on their own. Every person interacts frequently with the outside world, and the decisions made by a given person can have implications for many others. This kinship indicates while autonomy might be a valuable political concept, it is neither a psychological nor social one. People, whether they are professionals or patients, form a network of supports and constraints. Decisions made "autonomously" often echo through this network, changing the circumstances for other people.

## Abbreviations

LQT3: Long QT Syndrome type 3
TdP: Torsades de Pointes
LQTS: Long QT Syndrome
AMA: American Medical Association
NIH: National Institutes of Health
ICD: Implantable Cardiac Defibrillator
MEN: Multiple Endocrine Neoplasm/Medullary Thyroid Cancer
